# Efficiency of free thyroxine in predicting severity and mortality of patients with acute pancreatitis: A protocol for systematic review and meta-analysis

**DOI:** 10.1097/MD.0000000000024809

**Published:** 2021-02-19

**Authors:** Tao Cheng, Bo-Jie Xiao, Bo-Fu Liu, Tian-Yong Han, Hai-Fang Yu

**Affiliations:** a Emergency Department; b Laboratory of Emergency Medicine, West China Hospital; c Disaster Medical Center, Sichuan University, Chengdu, Sichuan, China.

**Keywords:** acute pancreatitis, free thyroxine, mortality, prognosis, severity

## Abstract

**Background:**

Previous studies suggest that free thyroxine may be used as a severity indicator of patients with acute pancreatitis (AP) in emergency department, helping determine the differential care of AP. However, there are no systematic reviews and the association between free thyroxine and AP is still not completely understood. Therefore, we will undertake a systematic review of the literature to summarize previous evidence regarding this topic, in order to clarify whether free thyroxine can help us pick out the mild AP cases.

**Methods:**

: We will search the EMBASE, Web of Knowledge, PubMed, ClinicalTrials.gov, and Cochrane Library from inception to Mar 2021 to retrieve relevant studies using the search strategy: (“free thyroxine”) AND (pancreatitis OR pancreatitides). Two authors independently judged study eligibility and extracted data. Heterogeneity will be examined by computing the Q statistic and *I*^*2*^ statistic.

**Results:**

: This study proved the efficiency of free thyroxine in predicting the severity of patients with AP.

**Conclusions:**

: This study will provide reliable evidence-based evidence for the clinical application of free thyroxine predicting the severity of patients with AP.

**Ethics and dissemination:**

Ethical approval is unnecessary as this protocol is only for systematic review and does not involve privacy data. The findings of this study will be disseminated electronically through a peer-review publication or presented at a relevant conference.

## 1 Introduction

Acute pancreatitis (AP) is a sudden inflammatory process in the pancreas^[1]^ and it is a common reason for hospitalization costing lots of resources annually.^[2]^ Severe AP cases are often associated with severe complications and high mortality,^[3,4]^ although mild AP cases can be successfully improved quickly but have unnecessarily long hospital stays.^[5]^ Therefore, it is necessary to predict the severity of AP earlier, for it is critical for determining early triage, aggressive resuscitation, and improving clinical outcomes of AP in high-risk patients. However, all prognostic systems in current use, such as Ranson criteria,^[6]^ Acute Physiology and Chronic Health Evaluation II (APACHE II),^[7]^ and Balthazar grade,^[8,9]^ involve many tests and are not convenient for clinical practice.^[10,11]^

Previous studies suggest that free thyroxine may be used as a severity indicator of patients with AP in emergency department, helping determine the differential care of AP.^[12–14]^ To explore whether free thyroxine can help us pick out the mild AP cases who can be successfully improved quickly and unnecessarily long hospital stays, we will undertake the systematic review and meta-analysis.

## 2 Methods and analysis

### 2.1 Registration

This meta-analysis protocol is based on the Preferred Reporting Items for Systematic Reviews and meta-analysis Protocols (PRISMA-P) statement guidelines. The PRISMA-P checklist for the protocol is provided in the PRISMAP-checklist. This protocol has been registered on International Prospective Register of Systematic Reviews database. The registration number was INPLASY202110088.

### 2.2 Eligibility criteria

The inclusion criteria for the study will include:

1. studies with patient age ≥18 years old, a minimum hospital stay of 24 hours, and a diagnosis of AP;
2. conference abstracts were only included when they provided adequate relevant information for assessment;
3. free thyroxine was used for the prediction of severity or mortality of patients with AP.

Exclusion criteria will include: age <18 years old, patients with chronic pancreatitis, pancreas carcinoma, thyroid disorders or history of thyroid surgery, and patients with incomplete data.

### 2.3 Searching strategy

**Figure 1:**
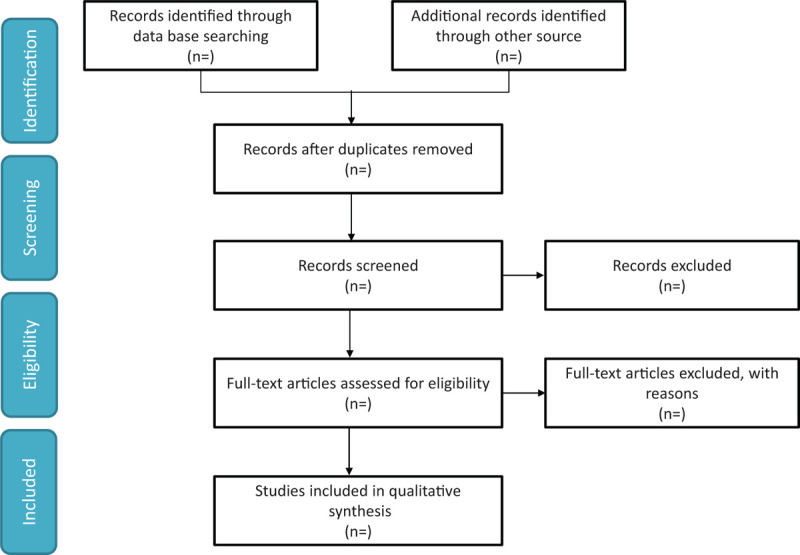
A flow diagram demonstrating the search strategy and study selection process for this study.

We will search the EMBASE, Web of Knowledge, PubMed, ClinicalTrials.gov, and Cochrane Library from inception to Mar 2021 to retrieve relevant studies using the search strategy: (“free thyroxine”) AND (pancreatitis OR pancreatitides). No language restrictions will be applied. We will also search citations of relevant primary and review. Authors of abstract in the meeting will be further searched in PubMed for potential full articles. To minimize the risk of publication bias, we will conduct a comprehensive search that included strategies to find published and unpublished studies. The research summary of the screening flow chart is shown in Figure 1.

### 2.4 Data extraction and risk of bias

Two reviewers will be employed the searching strategy respectively, by reading the papers and scoring them according to the QUADAS-2 checklist^[15]^ and Newcastle–Ottawa Quality Assessment Scale^[16]^; disagreement will be settled by a third opinion. Important information will be abstracted from the included articles in a standardized form by 2 reviewers. Important information include the name of the first author, publication year, publication country, type of study, study population, sample size, defined criteria of severe acute pancreatitis, outcomes studied (severity and mortality rate), and free thyroxine level. Risk of bias assessment will be carried out according to the Newcastle-Ottawa Scale (NOS) to rate the internal validity of the individual studies, and funnel plots will be constructed to assess the risk of publication bias.

### 2.5 Statistical analysis

All pairwise meta-analytic calculations will be performed with Review Manager software (RevMan) version 5.3 (Cochrane collaboration). Heterogeneity will be examined by computing the Q statistic and *I*^2^ statistic, and presence of reporting bias by visual inspection of funnel plots. Statistical significance was considered when the *P* value < .05.

## 3 Discussion

It is well known that severe AP cases are often associated with severe complications and high mortality.^[4,14,17]^ However, mild acute pancreatitis cases do not need long hospitalization,^[5]^ and even can be treated out of hospital to recover quickly, which could reduce the proportion of hospitalizations and therefore save more costs. All prognostic systems in current use, such as Ranson criteria,^[6]^ APACHE II,^[7]^ and Balthazar grade,^[8,9]^ have significant weaknesses for involving many tests and are not convenient. Abnormalities in thyroid function parameters have been described in different pathologies, including AP, where significant changes in serum levels of thyroid hormones have been demonstrated. Some studies have reported that free thyroxine can predict the severity of patients with AP, but the conclusion is controversial.^[12–14,18]^

To identify whether free thyroxine can predict the severity and mortality of AP, we will conduct the systematic review and meta-analysis. And this article is a protocol of the systematic review and meta-analysis, which presents the detailed description of review implement. The results of our review will be reported strictly following the PRISMA criteria and the review will add to the existing literature by showing compelling evidence and improved guidance in clinic settings.

## Acknowledgments

The authors would like to acknowledge the participants and their families for taking part in the study.

## Author contributions

**Conceptualization:** Tao Cheng, Bo-Jie Xiao, Bo-Fu Liu, Hai-Fang Yu.

**Data curation:** Tao Cheng, Bo-Fu Liu, Tian-Yong Han.

**Formal analysis:** Tao Cheng, Bo-Jie Xiao, Hai-Fang Yu, Tian-Yong Han.

**Funding acquisition:** Hai-Fang Yu.

**Investigation:** Tao Cheng, Bo-Jie Xiao, Bo-Fu Liu, Tian-Yong Han.

**Methodology:** Tao Cheng, Bo-Jie Xiao, Bo-Fu Liu, Hai-Fang Yu. Tian-Yong Han.

**Project administration:** Tao Cheng, Hai-Fang Yu, Bo-Fu Liu.

**Resources:** Tao Cheng, Bo-Jie Xiao, Tian-Yong Han.

**Software:** Tao Cheng, Bo-Jie Xiao, Tian-Yong Han, Hai-Fang Yu.

**Supervision:** Tao Cheng, Bo-Fu Liu, Hai-Fang Yu.

**Validation:** Tao Cheng, Bo-Jie Xiao, Tian-Yong Han, Bo-Fu Liu.

**Visualization:** Tao Cheng, Bo-Jie Xiao, Bo-Fu Liu, Hai-Fang Yu.

**Writing – original draft:** Tao Cheng, Bo-Jie Xiao, Tian-Yong Han, Bo-Fu Liu.

**Writing – review & editing:** Tao Cheng, Hai-Fang Yu.
